# Regulatory initiatives to reduce sugar-sweetened beverages (SSBs) in Latin America

**DOI:** 10.1371/journal.pone.0205694

**Published:** 2018-10-19

**Authors:** Paola Bergallo, Valentina Castagnari, Alicia Fernández, Raúl Mejía

**Affiliations:** 1 Law Department, Universidad Torcuato Di Tella, Ciudad Autónoma de Buenos Aires, Argentina; 2 University of California San Francisco, San Francisco, California, United States of America; 3 Centro de Estudios de Estado y Sociedad, Ciudad Autónoma de Buenos Aires, Argentina; University of Pennsylvania, UNITED STATES

## Abstract

**Background and objectives:**

Latin American (LA) countries have begun to adopt a variety of regulations targeting sugar-sweetened beverages (SSBs) for public health reasons. Our objective was to characterize the regulatory strategies designed to reduce SSB consumption over the last decade, and assess the available evidence on their enforcement and impact.

**Methods:**

We searched legal and public health databases for public and private SSBs regulations in 14 LA countries and then conducted a systematic review of the available literature. We tracked comparative variations in the type of body issuing the regulations, their scope, and binding status. We present data following a 5-category framework we named NUTRE that classifies SSBs regulations as: (1) restrictions to SSB availability in schools (N), (2) taxes and other economic incentives to discourage consumption (U), (3) restrictions on advertising and marketing (T), (4) regulations on government procurement and subsidies (R), and (5) product labeling rules (E).

**Results:**

Since 2006, 14 LA countries have adopted at least 39 public and private SSB regulatory initiatives across the NUTRE framework. Comprehensive efforts have only been approved by Chile, México and Ecuador, while the rest have comparatively few initiatives. 28 out of the 39 regulatory initiatives were passed by legislative and executive bodies; 11 initiatives represent self-regulatory undertakings by the beverage industries. An 86% (24/28) of public sector regulations are binding; 56% (22/39) contain explicit monitoring or evaluation methods; and 62% (24/39) provide for sanctions. Moreover, 23 regulations specify the body in charge of monitoring the new rules and standards.

**Conclusions:**

LA countries are targeting SSB consumption through a variety of mechanisms, particularly via restrictions to availability in schools and through taxes. Interdisciplinary evidence comparing alternative regulatory strategies is scarce, and few studies offer data on impact and implementation challenges. More evidence and further comparative assessments are needed to support future decision-making.

## Introduction

In the past two decades, non-communicable diseases (NCDs) levels have soared across Latin America (LA), in part due to the fastest growing obesity rates in the world [1]. Sugar sweetened beverages (SSBs), which combine high caloric content and minimal nutritional value, are arguably one of the most important drivers of the obesity epidemic [2, 3]. With consumption levels rocketing [2], SSBs account for an important portion of the daily caloric intake of poor communities, with numbers ranging from 10% [2] to as much as 23% [4] of total calorie consumption. Moreover, though SSBs sales are decreasing in some regions of the developed world, they are on the rise in LA and other developing areas [5, 6]. Joining a global trend toward reducing consumption on public health grounds [7, 8, 9], SSBs have become increasingly appealing targets of regulation throughout Latin America. Little is known, however, regarding the recent Latin American wave of regulatory initiatives focused on restricting the affordability, availability and acceptability of SSBs. Our research had two specific goals. First, to describe the regulatory strategies that LA countries have adopted to reduce SSB consumption. Second, to assess the available evidence on these new regulatory strategies, their enforcement challenges, and their impacts.

## Materials and methods

To achieve these aims, we conducted two types of literature searches. For both searches, we focused on governmental and industry initiatives concerning SSBs approved from 2008 to 2016. “Regulatory initiatives” here refer to public policies that limit the affordability, availability and/or acceptability of SSBs. Multiple initiatives may be associated with a single regulatory source, as a single piece of legislation may create several initiatives. Initiatives were included provided they had been formally adopted, irrespective of their implementation stage. The first search was focused on regulatory and legal materials and consisted of an exploratory search in Google Scholar, Hein Online, Wiley Online Library, JStor and Project Muse. This search was conducted in English and Spanish using the following keywords: soda OR soft drinks OR SSB AND regulation OR initiative OR law. Secondary sources retrieved were assessed to track relevant information to access primary source regulations (laws, resolutions, decrees, guidelines or self-regulatory initiatives).

We excluded government policy initiatives that were too vague or too general to affect the availability of SSBs, such as (a) broad food guidelines aimed at the general public (present in most countries), (b) broad nutrition education initiatives and (c) other unspecific initiatives that favor healthy eating or physical activity habits but make no explicit reference to SSBs.

The second search consisted of a systematic literature review of the research describing or assessing the operation of new SSB regulations and policy initiatives conducted in SciElo, Redalyc, JSTOR, Medline, BVS and LILACS for the period ranging from January 2007 to April 2017. The following key words were surveyed (both in English and in Spanish): soda OR sugar sweetened beverages OR SSB OR sugary drinks OR nutrition OR food AND obesity AND Latin America OR Argentina OR Barbados OR Brazil OR Chile OR Colombia OR Costa Rica OR Dominica OR Ecuador OR Salvador OR Mexico OR Peru OR Uruguay OR Venezuela AND regulation OR public policy OR self-regulation OR law OR tax OR incentive OR label* OR school OR subsidy OR advertising OR publicity OR commercialization OR public contract. Key words were considered when appearing in “full text” but country names were selected only when they appeared in the title. This distinction was made to filter irrelevant results, using only countries where our initial research had shown relevant results. Our review was limited to academic articles published in peer-reviewed journals. We did not independently evaluate the quality of the studies. Because this search aimed at assessing the content and application of the new policy initiatives, experimental or modelling studies were intentionally excluded. Two trained reviewers (VC and PB) read the titles of all the citations retrieved from the electronic database searches and removed those clearly unrelated to regulations., Abstracts were then reviewed to further exclude studies that did not meet inclusion criteria. At the third stage, the full articles were checked for eligibility with cross-checking by senior investigators. Reasons for exclusion were documented at each stage.

Data from the searches was extracted and categorized according to a 5-category framework we developed called NUTRE (Table 1), an acronym corresponding to Spanish words and inspired by the NOURISHING project of the World Cancer Research Fund [10]. The five categories are:

N: (*Niños y alimentación escolar*) Restrictions on the availability of SSBs in schools, where only restrictions directly referring to SSBs are considered.
U: (*Impuestos y subsidios*) Economic incentives affecting the affordability of SSBs, i.e. taxes or incentives with explicit public health objectives.
T: (*Trabas a publicidad dirigida a niños*) Regulations of advertising or other promotion activities regarding unhealthy food or drinks. These refer to restrictions directly dealing with food and/or drinks, excluding restrictions aiming to protect children in general.
R: (*Regulación de contratación pública*) Government procurement and/or public contracting restrictions.
E: (*Etiquetado frontal*) Front-of package (FOP) labelling strategies.

**Table 1:** NUTRE framework.

	Category description	Number of countries with public regulations	Number of countries with private-initiative regulations	Countries	Year of policy sanction
N	Restrictions on the sale of SSBs in schools.	7	2	Brazil	2006, 2009, 2014, 2016 (V)
				Chile	2012, 2015
				Colombia	2016 (V)
				Costa Rica	2013
				Ecuador	2009, 2010, 2014
				Mexico	2010
				Peru	2013, 2015
				Uruguay	2013
U	SSB taxes with public health objectives	5	N/A	Barbados	2015
				Chile	2014
				Dominica	2015
				Ecuador	2016
				Mexico	2013
T	Advertising/ promotion restrictions	9	8	Argentina	2008 (V)
				Bolivia	2016
				Brazil	2006, 2013 (V), 2014
				Chile	2012, 2013 (V), 2015
				Colombia	2013 (V)
				Costa Rica	2013
				Ecuador	2014
				El Salvador	2015 (V)
				Mexico	2009 (V), 2013
				Peru	2013
				Uruguay	2013, 2013 (V)
				Venezuela	2008 (V)
R	Government procurement restrictions	2	N/A	Brazil	2009
				Ecuador	2009
E	FOP labelling strategies	5	1	Bolivia	2016
				Chile	2012, 2015
				Colombia	2016 (V)
				Ecuador	2013
				Mexico	2013, 2015
				Venezuela	2015

“Years” refers to years where official norms were approved.

(V) indicates private-initiative regulations.

## Results

### SSBs regulatory initiatives

We found 36 legislative or executive national regulations and 11 industry guidelines from 14 LA countries that set out 39 SSB initiatives spanned across the NUTRE framework (Table 2). These 39 comprehensive efforts were mostly concentrated in a few nations, notably Chile, Mexico and Ecuador, while the rest had less comprehensive initiatives. Brazil’s regulations were mostly concentrated in advertising restrictions (T), schools (N), and public procurement initiatives (R). Ecuador is the only country that has enacted government regulations across all the NUTRE categories; industry has not adopted any private-initiative regulations in that country.

**Table 2:** Regulatory content of initiatives per category of the NUTRE framework (numbers refer to countries).

	N	U	T	R	E	Total
	Children	Taxes	Marketing	Government Procurement	Labelling	
**Type of regulation**						
Public	7	5	9	2	5	28
Private	2	0	8	0	1	11
**Binding status (for public regulations only n = 28)**						
Mandatory compliance	4	5	8	2	5	24
Voluntary compliance	1	0	0	0	0	1
Mixed	2	0	1	0	0	3
**Monitoring and evaluation methods**						
Yes	5	5	5	1	2	18
No	3	0	9	1	4	17
Mixed	1	0	3	0	0	4
**Sanctions**						
Yes	3	5	6	1	4	19
No	5		7	1	2	15
Mixed	1		4			5
**Monitoring body**						
Yes	5	5	7	1	2	20
No	3		8	1	4	16
Mixed	1		2			3

“Mixed” refers to regulations where application varies depending on the subject upon whom duties are applied.

Twenty-eight of those initiatives were passed by legislative and executive bodies, while 11 others were self-regulatory undertakings from beverage industries. Among the total regulatory initiatives sanctioned by governmental institutions, 24 imposed mandatory requirements, one contained non-mandatory guiding principles and 3 include mixed regulations where compliance is mandatory or not depending on the subject upon whom duties are applied. Roughly half the initiatives contain explicit monitoring or evaluation methods and a similar proportion provides for some form of sanction in case of lack of compliance. Moreover, 20 initiatives specify the body in charge of monitoring the new food and drink rules and standards.

### Evidence on implementation

**Fig 1:**
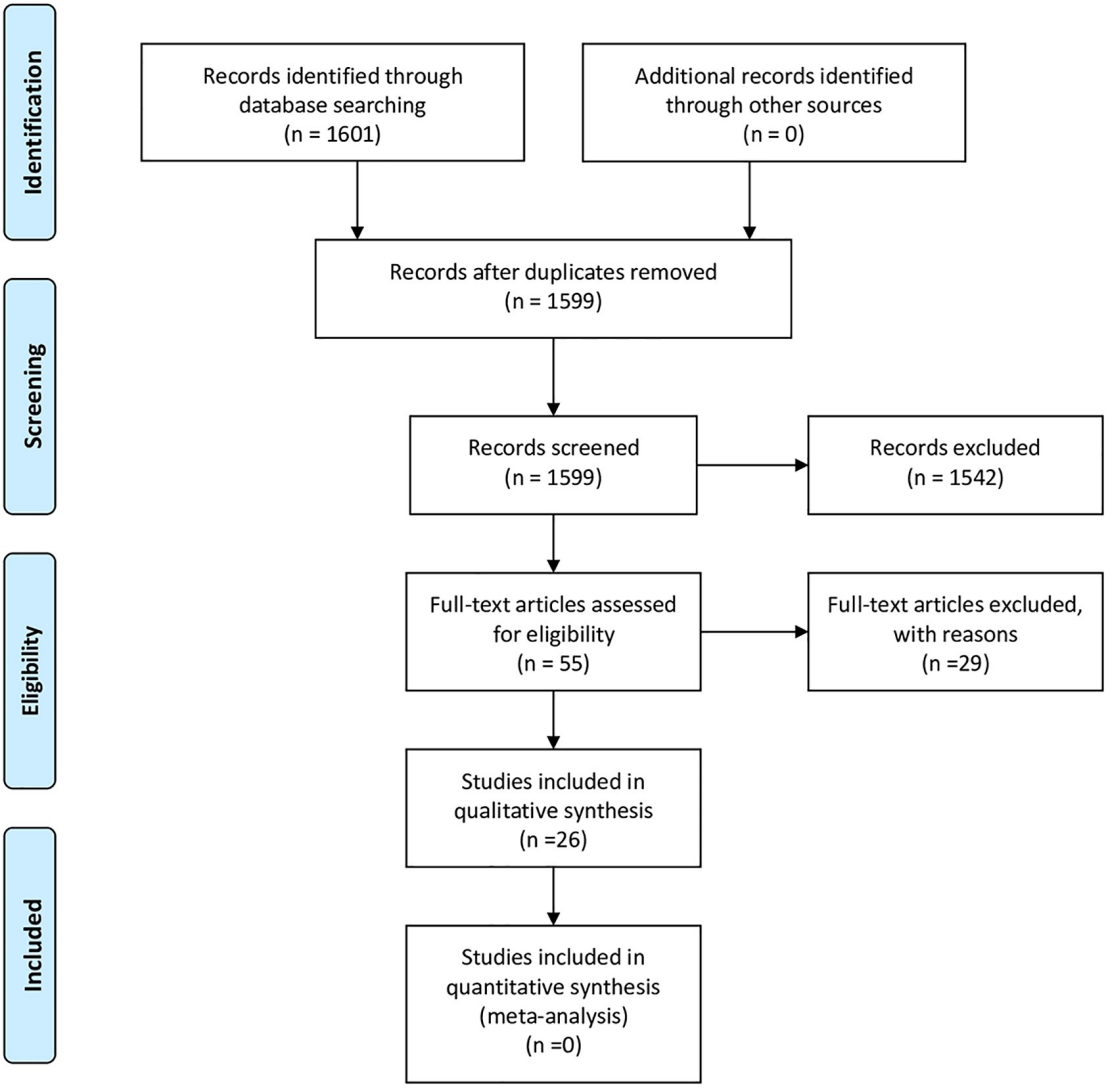
Prisma flowchart.

In our second search, focused on implementation, we 1601 articles were initially identified and titles and abstracts were screened, 55 articles were assessed in full-text (Fig 1). After discarding ineligible articles, the remaining 26 were classified based on whether they focused on describing regulatory initiatives (14), analyzing implementation of those initiatives (4) or appraising their impact (5). 3 articles focused both on describing policies and analyzing their implementation. The information retrieved from the literature review was used to (a) cross-check the regulatory sources collected as part of the first search and primarily used for the NUTRE framework, and (b) appraise the implementation of the initiatives. This data is analyzed in the next section.

## Findings in each NUTRE category

Using data on the regulatory initiatives classified in the NUTRE framework and the information retrieved from the literature review, we describe and critically assess the regulatory episodes corresponding to each category in the NUTRE framework as follows.

### Restrictions on the availability of SSBs in schools (N)

SSB consumption in schools is critical not only for the direct effect on children’s health, but also because schools have traditionally been crucial for companies to create brand awareness and build social norms [5, 11]. LA countries have become increasingly aware of the importance of the issue and SSB regulation efforts have intensified in the past few years (only Brazil and Ecuador regulated SSB availability before 2010 and five other countries have done so since).

However, public policies differ greatly in their origin and their content (Table 3). Brazil, Ecuador, Chile, Peru, and Uruguay have enacted national statutes via their legislative branches as well as executive-level decrees to regulate SSB availability in schools, whereas in Costa Rica and Mexico, restrictions have come from executive-level decrees only, suggesting less broad social consensus. All these regulations are mandatory. However, most of them (especially administrative regulations that are enacted by the executive branch via decrees or resolutions) do not provide for specific sanctions in case of non-compliance. This is the case, for instance, of the regulations approved in Ecuador. Mexico is the exemption, as its decree to ban drinks with over 10cal/250ml from schools (except on Fridays in secondary schools) imposes clear-cut sanctions for infringement.

**Table 3:** Restrictions on the sale of SSBs in schools. (V) refers to private-initiative regulations.

Country	Mandatory compliance	Restricted drinks [Categories adapted from [5]}			Coverage	
		Type	Max sugar content permitted /100ml	Restricts artificially sweetened beverages	Includes private schools	Includes high school
Brazil	No	All SSBs that exceed certain sugar content	"High in" sugar	No	Yes	No
Brazil (V)	Yes	All drinks except water, milk, 100% fruit juice	N/A	Yes	Yes	No
Chile	Yes	All SSBs that exceed certain sugar content	5g	No	Yes	No
Colombia (V)	Yes	All drinks except water, milk, 100% fruit juice, drinks with over 12% fruit and cereal-based drinks	N/A	Yes	Yes	No
Costa Rica	Mixed	All carbonated drinks and SSBs that exceed certain sugar content	6g	Yes	Yes (non-mandatory)	Yes
Ecuador	Yes	All SSBs that exceed certain sugar content	7.5g	No	Yes	Yes
Mexico	Yes	All drinks except water (primary school) // All drinks that exceed caloric content (10 cal/250ml)	N/A	Yes (primary school) // No (secondary school)	Yes	Yes
Peru	Yes	All SSBs that exceed certain sugar content	2.5g	No	Yes	Yes
Uruguay	No	All drinks except water, milk, 100% fruit juice	7.5g	Yes	Yes	Yes

Regulatory norms also vary in their scope and content, ranging from complete SSB bans to softer recommendations to avoid their sale. Prohibitions are usually based on a maximum content of added sugar; this varies by amount. The most stringent benchmark, in Peru, bans all drinks with over 2.5g sugar/100ml and the most permissive, in Ecuador and Uruguay bans drinks with over 7.5g/100ml, but parameters generally disallow full-calorie SSBs. Added sugar rules leave untargeted drinks such as 100% fruit juices that, although high in calories and a potential obesity prevention target, do not contain artificially added sugar. Only Mexico has established a strict maximum calorie content for drinks, virtually banning the sale of all drinks but water in secondary schools (while primary schools have an explicit water-only policy).

In 2016, beverage companies responded to the regulatory trend, adopting self-imposed commitments pledging to sell only water, drinks with over 12% fruit juice and cereal-based drinks in primary schools in Colombia [12] and only water, fruit juice, coconut water and dairy products in schools for children under 12 years (or mostly under 12) in Brazil [13]. These private-initiative pledges are actually more stringent in their content than official regulations. However, self-regulatory commitments have other limitations -especially as far as implementation is concerned—since there is no control over their enforcement and thus, they are often ignored [14].

Most SSB bans cover both public and private schools. Only Costa Rica makes a distinction, with a mandatory ban in public schools and softer recommendations for private schools [15]. The overall regional pattern differs from the trend in other parts of the world, where distinctions between public and private schools are more common [5]. Moreover, not all of the Latin American restrictions apply to secondary schools, where adolescents are sometimes considered mature enough to make their own choices and none applies explicitly to universities. Ecuadorean law applies to “educational establishments at all levels” [16], but it is unclear whether universities are included in this category. Inclusion of universities was an issue of much debate in both Chile (at congressional discussions) [17] and in Mexico (at the Supreme Court level) [18]. In both cases universities were ultimately considered not included within the regulations. Softer regulations were issued in Uruguay and Brazil, where guidelines contain specific recommendations not to offer SSBs at schools, but schools are not legally bound to comply.

There is a noteworthy lack of regulation of beverages containing artificial sweeteners in schools in the region. Only Mexico has set limits to the amount of artificial sweetener acceptable for drinks to be sold in schools and Costa Rica indirectly limits the availability of low calorie soft drinks by prohibiting all carbonated drinks. This is similar to the experiences from around the globe, where regulations consistently ban full-calorie beverages but have heterogeneous approaches regarding the limitation of low calorie sweeteners [5]. As the evidence on the long-term health effects of artificial sweeteners and their role in preventing weight gain continues to evolve [19], regulations may need to do so as well.

LA regulations also fail to encompass SSB sale in areas around schools, which might limit the efficiency of the proposed policies, since studies have described the associations between the number of food vendors around a school, the availability of processed and unhealthy food in the area, and children’s BMI [20, 21]. Despite their potential, restrictions to the sale of unhealthy foods in the vicinity of schools are relatively scarce not only in the region, but also around the globe. South Korea is a noteworthy exception, since it has established “Green Food Zones” 200 meters around schools, where the sale of junk foods and beverages is restricted [22]. Although it is too soon to measure the impact of that experience on health, some indicators seem promising [23]. In the United States, some cities have established restrictions to the establishment of fast-food restaurants in the proximity of schools [24], but these efforts are few somewhat scattered.

There is little research evidence on the challenges faced in the implementation of school SSB regulations. Roughly, half of the analyzed regulations refer to monitoring and/or evaluation schemes *in the books*, but provide little information on their application in practice.

Available studies focus mainly on Mexico, where the National Agreement for Healthy Nutrition (ANSA, for its Spanish acronym) and its subsequent “General Guidelines to regulate the distribution of food and drinks at schools” [25] have reportedly encountered strong implementation barriers [4, 26, 27, 28]. These limitations are mainly related to structural weaknesses [28, 29], especially the lack of a strong official implementation strategy, that takes into account education regarding the policies, operational training, cultural barriers; and strategies to overcome the economic pressures of school beverage vendors [29]. The two studies assessing this issue in Mexico at the local level concluded that guidelines are generally not followed [29, 30]. Strong industry opposition [30] was noted as a crucial obstacle to the implementation of these regulations [4] and proved especially strong in the process of enacting Mexico’s General Guidelines on the distribution of food and drinks at schools [11]; which were weakened due to industry pressure [4, 11].

None of the studies captured in the literature review reports on an evaluation of the impact of national school-based initiatives carried out in Latin America. However, at least one study has reviewed evaluations of other school-based initiatives carried out at the local level and concluded that local level initiatives can have a positive impact on weight gain and should thus be recommended [31]).

### Use of economic incentives that affect the affordability of SSBs (U)

Taxes on unhealthy food or drink have been promoted by the World Health Organization as potentially effective measures to reduce consumption [32]. Taxes can be levied in the forms of excise, sales or value-added (VAT) taxes [33], although experts usually advocate for excise taxes [3], as they increase shelf price and are easier to collect (reducing transaction costs and the likelihood of tax evasion) [29]. Tax regulations also vary in terms of the kind of product taxed and the applicable rate (Table 4). Fixed tax rates should foresee the possible impact of inflation, which could dilute the effect of the tax. This is an important issue in the acute inflationary context of the region, and both countries which use this type of rate (Ecuador (for SSBs with over 25g sugar per liter) and Mexico) have anticipated it. While in Ecuador the tax is updated annually according to the variation of the Consumer Price Index [34], in Mexico it is updated only when the accumulated increase in the Consumer Price Index exceeds 10% [35]. Only taxes which explicitly refer to health purposes were included in the NUTRE framework.

**Table 4:** SSB taxes in Latin American countries.

Country	Tax				Beverages taxed		
	Type of tax	Type of rate	Rate	Adjusts to inflation	Type of beverage	Includes artificially sweetened drinks	Includes energy drinks
Mexico	Excise	Fixed	$1/liter	Yes	All SSBs	No	Yes
Barbados	Excise	% of price	10%	N/A	All SSBs	No	Yes
Dominica	Excise	% of price	10%	N/A	All SSBs	No	Yes
Ecuador	Excise	% of price		N/A	Carbonated beverages with ≤25g sugar/liter	Yes	Yes
	Excise	Fixed	us$0,18 /100g sugar	Yes	SSBs with ≥25g sugar/liter	No	No
Chile	VAT	% of price	10%	N/A	Non- alcoholic drinks with added coloring, flavoring or sweeteners	Yes	Yes
	VAT	% of price	18%	N/A	SSBs ≥15g sugar/240ml	No	Yes

Tax efforts usually require legislative change and thus demand levels of social agreement that are not easy to achieve. Moreover, health-related taxes have been vigorously opposed by the SSB industry using a wide array of arguments, including, among others, the potential for job and other economic losses, disproportional impact on the poorest populations, and illegitimacy of government market intervention. The industry’s strong lobbying practices have proven successful in Colombia [36] and in Argentina, where proposals for a tax on SSBs were defeated in Congress in 2016 and 2017 respectively [37].

Four countries in the region have implemented excise taxes on SSBs and Chile raised its VAT 5% for SSBs with more than 15g sugar/240ml (while decreasing the VAT for drinks without sugar). Mexico is the most salient case, not only because it was the first country to implement such a tax in 2013, but also because the process to pass the 1 peso/liter tax to all SSBs was fierce on both sides of the struggle and gained massive global media attention. Mexico’s experience could therefore prove to be a key case-study to determine the impact of the tax on SSB consumption, thus possibly encouraging other countries to use fiscal policies to reduce SSB and junk food consumption [48]. Four out of six of the studies we retrieved as part of our literature review refer specifically to Mexico’s SSB and junk food tax. One of these studies [38] analyzed the tax pass-through rate (i.e. whether the tax is passed on to consumers via prices) in rural areas and concluded that the tax had not been fully transferred to prices (at least through December 2014). The other three papers dealing with Mexico’s tax policies [39, 40, 41] evaluate the effects of the tax on consumption of SSBs or junk food. As far as SSB consumption goes, both studies [40, 41] (by the same lead author) concluded that consumption decreased in the first and second year after the implementation of the tax and that the drop in consumption appears to be increasing. These findings are consistent with the research in other parts of the world [42, 43]. All these studies, however, have certain important limitations, most notably the impossibility of determining whether consumption has declined solely on account of the tax implementation or has been affected by other factors (e.g. increased public health education, campaigns, etc.). There are as yet no studies assessing the impacts of the recent taxes on health.

It should be noted that another study was published after our review ended, examining the trends in beverage prices in Barbados after the introduction of the tax on SSBs [44]. Although preliminary, this study shows that, following the implementation of the tax, the prices of SSBs increased 5.9% in average, whereas prices for non-SSBs remained mostly flat. This evidence shows that the tax is being passed onto prices, although not fully. This is consistent with the Mexican experience (although the Mexican case is even more promising, since the tax was fully passed onto prices in urban areas), and points to the fact that in both cases the tax is indeed being passed onto prices, the first step for it to have the desired effect on consumers. It should be noted that, because this is a subject under constant review, it is likely that other articles will appear before publication.

The studies also reveal other limitations in LA tax reforms. None of the region’s taxes reach the 20% price increase recommended by experts for consistent impact on consumption and hence on health [32, 33]. It should also be noted that earmarking tax revenue to specific health-related objectives (public awareness campaigns, prevention programs, obesity treatment, etc.) has been shown to increase public support of these taxes in other countries [2, 3, 33], but it in some LA countries, e.g. Mexico, this type of earmarking is not legally permitted.

### Regulations to advertising or other forms of promotion of unhealthy food and/or drinks (T)

Restrictions to the advertising and promotion of unhealthy food or drinks are by far the most widespread regulations in Latin America. Not only have nine countries taken official action in this direction, but industry-led initiatives have also been prolific.

Regulatory initiatives have adopted three basic forms: guidance, restrictions or messaging (Table 5). Guidance is the most common, especially in industry pledges. It provides good-practice standards when food and drink advertising is directed to children, usually through generic commitments not to motivate unhealthy habits (such as overeating or sedentary lifestyles) or pledges not to exploit children’s innocence so as to increase sales. However, clear rules are usually lacking, and the specific content of these standards can be interpreted broadly. Moreover, pre-established monitoring strategies and sanctions are rare (although not inexistent). Finally, private committees whose objectivity is dubious are the usual monitors of industry pledges.

**Table 5:** Distribution of public and private initiatives across the NUTRE framework in Latin America.

Country	N		U		T		R		E	
	Children		Taxes		Advertisement		Government Procurement		Labelling	
	Public	Private	Public	Private	Public	Private	Public	Private	Public	Private
Argentina						x				
Barbados			x							
Bolivia					x				x	
Brazil	x	x			x	x	x			
Chile	x		x		x	x			x	
Colombia		x			x	x				x
Costa Rica	x				x					
Dominica			x							
Ecuador	x		x		x		x		x	
El Salvador						x				
Mexico	x		x		x	x			x	
Peru	x				x					
Uruguay	x				x	x				
Venezuela						x			x	

“Public” refers to government initiatives and “Private” indicates private-initiative regulations.

Restrictions also differ in their content and scope. Comparisons among countries are somewhat problematic because variations occur at different levels including, among others: (a) the definition of publicity/promotion (does it include free distribution? Sponsorship? Special promotions?); (b) media covered by the regulations (social media is an especially controversial issue); (c) the scope of age-protection (which ranges from ages 12 to 18); (d) the conditions under which advertisements are considered to be targeted at children (by measuring audience, by the use of famous characters, by the use of colors, etc.); (e) standards used to define “unhealthy food and drinks” (whether they are generically defined or apply specific rules regarding maximum sugar content); and (f) sanctions for non-compliance.

Overall, Chile has the strictest restriction, having established a full prohibition on advertising food and drinks that are considered “high” in certain “critical nutrients” for children under 14 [45]. Chilean regulation does not specify the media covered, although the wording of the law includes a very broad definition of publicity, which suggests it covers media in all of its forms. Furthermore, the country has explicitly prohibited free distribution and the use of “commercial hooks” to attract children (including the use of famous characters, games, etc.). It has also completely banned the advertisement of unhealthy food and drinks on TV and in cinemas from 6 am to 10 pm (regardless of the targeted audience), and only allows it in public events, such as sports or cultural events, in exceptional cases.

In contrast, Mexico has also prohibited advertising unhealthy food and drinks to children, but its restrictions cover only TV and cinemas [46]. The time frame is more permissive and certain program exclusions (such as sports events or soap operas) further narrow children’s protection. In a different approach, Brazil, considers all publicity targeting children (food-related or otherwise) “abusive” [47], although the practical implications of such a statement would require further case-by-case analysis.

Restrictions usually anticipate penalties for non-compliance, but monitoring strategies are less frequent. Private initiatives in Brazil and Colombia are wide-ranging in their content, pledging not to advertise to children under 12, but include no specifications regarding implementation efforts [12, 13].

Guidance or restrictions to advertising within the school environment are also common in Latin America. Restrictions usually imply a full ban of advertisement in that setting and, with the exception of Costa Rica’s ban that is not mandatory in private schools [15], they all apply to both public and private schools at all levels. However, only Chile has designed some sort of implementation strategy and Chile and Ecuador are the only countries to impose sanctions for non-compliance as part of these regulations.

Finally, some countries mandate food or drink advertisements to include messages to either promote healthy lifestyles (Bolivia and Chile), warn about the potential health effects of certain products (Brazil and Peru) or do both (Ecuador and Mexico). Argentina attempted to include such messaging, but the law was vetoed by the President in 2008. Brazil’s messaging regulation was also suspended after a suit questioned the rules before the courts [6].

There is very little information regarding the implementation and/or impact of these advertising regulations. Our review of the literature retrieved only one study [14] addressing the issue directly by empirically observing the operation of pledges regarding food and drink advertisements by signatory companies in Mexico. That study found that, despite the adoption of industry pledges, Mexican children are heavily exposed to unhealthy food and drink ads on TV, as 75% of these are directly or indirectly aimed at them.

### Government procurement and/or public contracting restrictions (R)

Restrictions to the kind of beverages that can be purchased using government funds are the least common forms of regulation in the region, although their potential to affect consumption patterns could prove enormous [6]. LA governments often provide food-related assistance either directly (in public school cafeterias, for example) or indirectly (through subsidies to the poor), therefore, restrictions of the kind of food or drinks that can be purchased are likely to affect many in the most vulnerable sectors of the population. Egalitarian arguments aside, there are strong arguments in favor of restrictions to the purchase of SSBs, given their low nutritional value and their harmful health effects, which contradict the very spirit of government assistance.

Only Brazil and Ecuador have taken action in this area and both have done so through national legislation. Brazil has prohibited the purchase of drinks low in nutritional quality using resources from the National Education Development fund, which finances the national school food plan. It has also stated that a minimum of 30% of the expenditures must be used to purchase food from local farmers. Ecuador has applied a broader regulation (not restricted to the school environment), banning the distribution and use of products low in nutritional quality in food aid programs.

Although narrower in scope, Brazil’s legislation has a more detailed implementation strategy, since provincial states are obliged to account for their expenses at the end of each school year and can be denied further assistance if their expenditure has not complied with the purchasing standards. In contrast, Ecuador has not determined legal sanctions for breaches of the applicable restrictions, although it has created an *ad hoc* entity to promote the practical enforcement of the law.

No research on the implementation or the impact of contracting regulations was found in our literature review.

### Front-of package labelling strategies (E)

Front-of-package (FOP) labelling strategies aim at improving point-of-purchase consumer information to encourage healthier buying decisions and product reformulation. Although most countries in the region apply some sort of mandatory nutritional labeling in the back of food packages, compulsory FOP labelling is still relatively uncommon. The first country to impose a FOP labelling regulation was Chile in 2012 and four other countries have followed since. Most of these regulations have been approved through administrative resolutions and only Chile and Bolivia have enacted laws specially focused on FOP labels. Industry-led initiatives are also scarce at the national level, but Coca Cola has implemented a private-initiative international pledge to include calorie, sugar, fat and salt content in all its product labels. Moreover, within Colombia, the Drink Industry Chamber (*Cámara de la Industria de Bebidas de la Asociación de empresarios de Colombia*) has also committed to implement a FOP label in drinks [12] (although the pledge does not specify what information will be included).

The label design required by FOP regulations differs among countries. Bolivia and Ecuador have chosen a traffic-light signal to graphically warn consumers about high levels of sugar, calories, fat and salt. Although some studies have suggested that this label design is the one which most consistently helps consumers identify healthier products [48,49], recent evidence has suggested that clarity and simplicity might be the most important features of food labeling systems [50, 56]. This may make warning labels more promising than traffic-light labels [51] and GDA [52]. However, more research comparing different strategies is needed before reaching robust conclusions. The traffic-light label design was initially suggested in Chile [17], but the proposal was overturned and changed into an octagonal black and white warning sign that alerts consumers when products are “high in” certain nutrients. Finally, Venezuela regulated SSB labels in particular, applying mandatory warning messages stating the risks of consumption. Corporations have traditionally favored labels that refer to Guideline Daily Amounts (GDA) as opposed to warning signs. GDA labelling is the one adopted by the Coca Cola pledge and the one applied in Mexico.

Most LA countries have adopted negative FOP labelling, where consumers are warned against the consumption of certain products as opposed to encouraged to purchase healthier options. Only Mexico has designed a positive distinctive sign (called “*sello nutrimental*”) to identify healthier consumer alternatives, which can be used by food companies only after official authorization is granted. The use of this sign is voluntary and was designed to promote foods that meet certain nutritional criteria, which is why it can only be used after authorities certify that the product meets those criteria and is therefore eligible to be promoted accordingly.

Regulations also vary in the nutrient profiling schemes they use to determine which food and drinks will carry a special label, although most apply a specific reference amount of sugar/100 ml for SSBs. Although simple and widely used, this kind of scheme has the disadvantage of not considering real serving sizes and conditions [53].

There is little information regarding the implementation of labelling strategies in Latin America. Regulations mostly lack implementation and monitoring structures, although formal sanctions are referred to in Chile, Mexico, Ecuador and Venezuela. Research from around the globe provides inconclusive evidence as to consumer comprehension of labels and whether labelling strategies effectively help change consumption habits and under which circumstances [6, 7, 48, 54, 55, 56,57]. Studies on the impact of the LA labelling efforts were not found in our review of the available literature and are urgently needed.

## General limitations

Our study has some limitations that should be acknowledged. The methodology used to retrieve regulatory initiatives was to some extent opportunistic, since systematized evidence regarding legal regulations on SSBs in the region is scarce and/or not thorough. Hence, although we attempted to capture all the relevant initiatives, some might have been left uncovered. We also used the systematic literature review to cross-check our information but given the scarcity of academic research on these recent regulations, this search provided little additional information. In order to aid future research, we have built a database with all the legal regulations retrieved and posted them online in the web of the B.A.S.T.A. project available at http://proyectobasta.esy.es.

## Conclusion

Latin American countries are attempting to decrease SSB consumption through the use of various types of regulatory initiatives. We have analyzed each type of regulation separately for practical purposes. However, it should be highlighted that any attempt to seriously tackle the obesity epidemic should address the issue in multiple dimensions, since any single measure would most likely prove insufficient [58]. Chile, Ecuador and Mexico’s experiences are outstanding in this regard, as they have implemented a package of interventions across the NUTRE categories. Ecuador and Chile’s cases are especially salient because their labelling schemes help identify products that are banned from being sold or publicized in schools and from being advertised to children. Mexico, by contrast, lacks a comprehensive strategy linking its FOP labelling design with the application of other regulations. This means that additional regulations are necessary to identify the products that can (or cannot) be sold in schools or advertised, making it potentially challenging to identify infringements.

Chile’s case is especially salient because of its innovative, wide ranging policies which have earned the country global recognition. Its labelling design is currently regarded as the most promising in the world and is being used as a model and discussed by other countries in the region, including Peru, Uruguay, Brazil and Argentina. Chile’s path is noteworthy because its evidence-based framework law simultaneously covers most of the policies that have been recommended to tackle the obesity epidemic on all fronts, which is why it is being observed auspiciously.

SSB industry resistance has been fierce [4, 30, 53] and is unlikely to cease given the decrease in SSB consumption rates in developed-countries and the increased market share devoted to Latin America. Therefore, governments and public-health advocates should anticipate the industry’s well-resourced, well-organized strategies.

Greater efforts should be made to build consensus across multiple constituencies, both in the process of discussing reforms and in implementation stages. There is broad agreement regarding the need for further integration between the public sector (at a national and local level) and the private sector, academia and civil society organizations to secure successful obesity prevention reforms [4, 30, 59, 60, 61]. Furthermore, there has often been a lack of public participation in obesity-related reform processes in the region [4, 53, 60], which is likely to be an obstacle for the successful enforcement of new food and drink regulations.

Information regarding the actual implementation of reforms, the human and financial resources required and, eventually, their impact, is scarce for most of the new regulatory initiatives undertaken across LA [61]. As the analysis of the NUTRE framework shows, regulations (mandatory and otherwise) often lack clear implementation, monitoring and evaluation strategies, key factors in the success of legal reforms [61, 62, 63]. Evidence also accounts for a lack of training and information of people meant to apply the new rules and, consequently, resistance to change [29]. Much more research is needed to guide government and public health advocates seeking to create effective SSB policies.

## Supporting information

S1 File PRISMA checklist. (DOC)
